# The behaviour change wheel: A new method for characterising and designing behaviour change interventions

**DOI:** 10.1186/1748-5908-6-42

**Published:** 2011-04-23

**Authors:** Susan Michie, Maartje M van Stralen, Robert West

**Affiliations:** 1 Research Dept of Clinical, Educational, and Health Psychology, University College London, 1-19 Torrington Place, London WC1E 7HB, UK; 2 VU University Medical Center, EMGO Institute for Health and Care Research, Van der Boechorststraat 7, 1081 BT Amsterdam; 3 Health Behaviour Research Centre, University College London Epidemiology and Public Health, London, UK

## Abstract

**Background:**

Improving the design and implementation of evidence-based practice depends on successful behaviour change interventions. This requires an appropriate method for characterising interventions and linking them to an analysis of the targeted behaviour. There exists a plethora of frameworks of behaviour change interventions, but it is not clear how well they serve this purpose. This paper evaluates these frameworks, and develops and evaluates a new framework aimed at overcoming their limitations.

**Methods:**

A systematic search of electronic databases and consultation with behaviour change experts were used to identify frameworks of behaviour change interventions. These were evaluated according to three criteria: comprehensiveness, coherence, and a clear link to an overarching model of behaviour. A new framework was developed to meet these criteria. The reliability with which it could be applied was examined in two domains of behaviour change: tobacco control and obesity.

**Results:**

Nineteen frameworks were identified covering nine intervention functions and seven policy categories that could enable those interventions. None of the frameworks reviewed covered the full range of intervention functions or policies, and only a minority met the criteria of coherence or linkage to a model of behaviour. At the centre of a proposed new framework is a 'behaviour system' involving three essential conditions: capability, opportunity, and motivation (what we term the 'COM-B system'). This forms the hub of a 'behaviour change wheel' (BCW) around which are positioned the nine intervention functions aimed at addressing deficits in one or more of these conditions; around this are placed seven categories of policy that could enable those interventions to occur. The BCW was used reliably to characterise interventions within the English Department of Health's 2010 tobacco control strategy and the National Institute of Health and Clinical Excellence's guidance on reducing obesity.

**Conclusions:**

Interventions and policies to change behaviour can be usefully characterised by means of a BCW comprising: a 'behaviour system' at the hub, encircled by intervention functions and then by policy categories. Research is needed to establish how far the BCW can lead to more efficient design of effective interventions.

## Background

Improving the implementation of evidence-based practice and public health depends on behaviour change. Thus, behaviour change interventions are fundamental to the effective practice of clinical medicine and public health, as indeed they are to many pressing issues facing society. 'Behaviour change interventions' can be defined as coordinated sets of activities designed to change specified behaviour patterns. In general, these behaviour patterns are measured in terms of the prevalence or incidence of particular behaviours in specified populations (*e.g*., delivery of smoking cessation advice by general practitioners). Interventions are used to promote uptake and optimal use of effective clinical services, and to promote healthy lifestyles. Evidence of intervention effectiveness serves to guide health providers to implement what is considered to be best practice (for example, Cochrane reviews, NICE guidance). While there are many examples of successful interventions, there are also countless examples of ones that it was hoped would be effective but were not [[1], e.g. [2,3]]. To improve this situation, and to improve the translation of research into practice, we need to develop the science and technology of behaviour change and make this useful to those designing interventions and planning policy.

The process of designing behaviour change interventions usually involves first of all determining the broad approach that will be adopted and then working on the specifics of the intervention design. For example, when attempting to reduce excessive antibiotic prescribing one may decide that an educational intervention is the appropriate approach. Alternatively, one may seek to incentivise appropriate prescribing or in some way penalise inappropriate prescribing. Once one has done this, one would decide on the specific intervention components. This paper examines this first part of this process. We and others are also working on how one identifies specific component 'behaviour change techniques' [4,5].

In order to identify the type or types of intervention that are likely to be effective, it is important to canvass the full range of options available and use a rational system for selecting from among them. This requires a system for characterising interventions that covers all possible intervention types together with a system for matching these features to the behavioural target, the target population, and the context in which the intervention will be delivered. This should be underpinned by a model of behaviour and the factors that influence it.

Interventions are commonly designed without evidence of having gone through this kind of process, with no formal analysis of either the target behaviour or the theoretically predicted mechanisms of action. They are based on implicit commonsense models of behaviour [6]. Even when one or more models or theories are chosen to guide the intervention, they do not cover the full range of possible influences so exclude potentially important variables. For example, the often used Theory of Planned Behaviour and Health Belief Model do not address the important roles of impulsivity, habit, self-control, associative learning, and emotional processing [7].

In addition, often no analysis is undertaken to guide the choice of theories [8]. Useful guidance from the UK Medical Research Council for developing and evaluating complex interventions advocates drawing on theory in intervention design but does not specify how to select and apply theory [9]. It should also be noted that even when interventions are said to be guided by theory, in practice they are often not or are only minimally [10].

Thus, in order to improve intervention design, we need a systematic method that incorporates an understanding of the nature of the behaviour to be changed, and an appropriate system for characterising interventions and their components that can make use of this understanding. These constitute a starting point for assessing in what circumstances different types of intervention are likely to be effective which can then form the basis for intervention design.

There exists a plethora of frameworks for classifying behaviour change interventions but an informal analysis suggests that none are comprehensive and conceptually coherent. For example, 'MINDSPACE' an influential report from the UK's Institute of Government, is intended as a checklist for policymakers of the most important influences on behaviour [11]. These influences provide initial letters for the acronym MINDSPACE: messenger, incentives, norms, defaults, salience, priming, affect, commitment, and ego. The framework does not appear to encompass all the important intervention types. Moreover, the list is a mixture of modes of delivery (*e.g*., messenger), stimulus attributes (*e.g*., salience), characteristics of the recipient (*e.g*., ego), policy strategies (*e.g*., defaults), mechanisms of action (*e.g*., priming), and related psychological constructs (*e.g*., affect). In that sense it lacks coherence. The report recognises two systems by which human behaviour can be influenced -- the reflective and the automatic -- but it focuses on the latter and does not attempt to link influences on behaviour with these two systems.

A second example comes from the Cochrane Effective Practice and Organisation of Care Review Group (EPOC)'s 2010 taxonomy [12]. This categorises interventions to change health professional behaviour into professional, financial, organisational, or regulatory, covering many of the key intervention types. However, the categories are very broad and within each is a mixture of different types of interventions at different conceptual levels. For example, 'professional' includes individual behaviour (distributing educational materials) and organisational interventions (local consensus processes); 'financial' includes individual and organisational incentives and environmental restructuring (changing the available products); 'organisational' includes input (changing skill mix), processes (communication) and effects (satisfaction of providers); and 'regulatory' includes legal (changes in patient liability) and social influence (peer review). Professional, financial, and organisational interventions are found across all categories.

Aside from specific frameworks, there are some broad distinctions that have been widely adopted. One such distinction is between population-level and individual-level interventions [13]. While superficially appealing, there are many interventions that this distinction cannot readily classify and it has not been possible to arrive at a satisfactory definition of the distinction that does not contain inconsistencies. For example, if wide reach is a feature of population level interventions, routine general practitioner (GP) smoking assessment and advice (given to all patients) should fall into that category; yet it is delivered specifically to individuals and can be tailored to those individuals. Indeed, the NHS Stop Smoking Services might be considered a typical case of individual-level interventions, but they reach more than 600,000 smokers each year [14]. We do not consider these broad distinctions further in this paper.

It appears that most intervention designers do not use existing frameworks as a basis for developing new interventions or for analysing why some interventions have failed while others have succeeded. One reason for this may be that these frameworks do not meet their needs. In order to choose the interventions likely to be most effective, it makes sense to start with a model of behaviour. This model should capture the range of mechanisms that may be involved in change, including those that are internal (psychological and physical) and those that involve changes to the external environment. In general, insufficient attention appears to be given to analysing the nature of behaviour as the starting point of behaviour change interventions [15], a notable exception being intervention mapping [16]. 'Nature of the behaviour' was identified as one of 12 theoretical domains of influence on implementation-relevant behaviours [9]. Whilst this framework of 12 theoretical domains has proved useful in assessing and intervening with implementation problems [9], the domain of behaviour has remained under-theorised and therefore underused in its application.

There are a number of possible objections to attempting to construct the kind of behavioural model described and link this to intervention types. The most obvious criticism is that the area is too complex and that the constructs too ill-defined to be able to establish a useful, scientifically-based framework. Another is that no framework can address the level of detail required to determine what will or will not be an effective intervention. The response to this is twofold: these are empirical questions and there is already evidence that characterising interventions by behaviour change techniques (BCTs) can be helpful in understanding which interventions are more or less effective [6,17]; and not to embark on this enterprise is to give up on achieving a science of behaviour change before the first hurdle and condemn this field to opinion and fashion.

To achieve its goal, a framework for characterising interventions should be comprehensive: it should apply to every intervention that has been or could be developed. Failure to do this limits the scope of the system to offer options for intervention designers that may be effective.

Second, the framework needs to be coherent in that its categories are all exemplars of the same type of entity and have a broadly similar level of specificity. Thus, categories should be from a super-ordinate entity (*e.g*., function of the intervention), and the framework should not include some categories that are very broad and others very specific. A beautiful example of an incoherent classification system is the Ancient Chinese Classification of Animals: 'those that belong to the Emperor, embalmed ones, those that are trained, suckling pigs, mermaids, fabulous ones, stray dogs, those that are included in this classification, those that tremble as if they were mad, innumerable ones, those drawn with a very fine camel's hair brush, others, those that have just broken a flower vase, and those that resemble flies from a distance' (Luis Borges 'Other Inquisitions: 1937-1952').

In addition, the categories should be able to be linked to specific behaviour change mechanisms that in turn can be linked to the model of behaviour. These requirements constitute three criteria of usefulness that can be used to evaluate the framework: comprehensiveness, coherence, and links to an overarching model of behaviour. We limited the criteria to those we considered to form a basis for judging adequacy. There are others, *e.g*., parsimony, that are desirable features but do not lend themselves to thresholds. Other criteria can be used to evaluate its applicability, *e.g*., reliability, ease of use, ease of communication, ability to explain outcomes, usefulness for generating new interventions, and ability to predict effectiveness of interventions

In light of the above, this paper aims to:

1. Review existing frameworks of behavioural interventions to establish how far each meets the criteria of usefulness, and to identify a comprehensive list of intervention descriptors at a level of generality that is usable by intervention designers and policy makers.

2. Use this list to construct a framework of behaviour change interventions that meets the usefulness criteria listed above.

3. Establish the reliability with which the new framework can be used to characterise interventions in two public health domains.

## Methods

Prior to reviewing the literature on intervention frameworks, we needed to establish a set of criteria for evaluating their usefulness. Following this, our method involved three steps: a systematic literature review and evaluation of existing behaviour change intervention frameworks, development of a new framework, and a test of the reliability of the new framework.

### Establishing criteria of usefulness

From the analysis set out in the Introduction, we established three criteria of usefulness:

1. Comprehensive coverage -- the framework should apply to every intervention that has been or could be developed: failure to do this limits the scope of the system to offer options for intervention designers that may be effective.

2. Coherence, *i.e*., categories are all exemplars of the same type and specificity of entity.

3. Links to an overarching model of behaviour.

We use the term 'model' here in the sense defined in the Oxford English Dictionary: 'a hypothetical description of a complex entity or process.' For the overarching model of behaviour, we started with motivation, defined as: brain processes that energize and direct behaviour) [18]. This is a much broader conceptualisation than appears in many discourses, covering as it does basic drives and 'automatic' processes as well as choice and intention.

Our next step was to consider the minimum number of additional factors needed to account for whether change in the behavioural target would occur, given sufficient motivation. We drew on two sources representing very different traditions: a US consensus meeting of behavioural theorists in 1991 [19], and a principle of US criminal law dating back many centuries. The former identified three factors that were necessary and sufficient prerequisites for the performance of a specified volitional behaviour: the skills necessary to perform the behaviour, a strong intention to perform the behaviour, and no environmental constraints that make it impossible to perform the behaviour. Under US criminal law, in order to prove that someone is guilty of a crime one has to show three things: means or capability, opportunity, and motive. This suggested a potentially elegant way of representing the necessary conditions for a volitional behaviour to occur. The common conclusion from these two separate strands of thought lends confidence to this model of behaviour. We have built on this to add non-volitional mechanisms involved in motivation (*e.g*., habits) and to conceptualise causal associations between the components in an interacting system.

**Figure 1:**
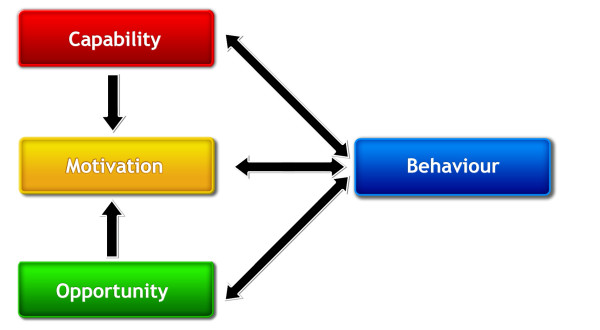
**The COM-B system - a framework for understanding behaviour**.

In this 'behaviour system,' capability, opportunity, and motivation interact to generate behaviour that in turn influences these components as shown in Figure 1 (the 'COM-B' system). Capability is defined as the individual's psychological and physical capacity to engage in the activity concerned. It includes having the necessary knowledge and skills. Motivation is defined as all those brain processes that energize and direct behaviour, not just goals and conscious decision-making. It includes habitual processes, emotional responding, as well as analytical decision-making. Opportunity is defined as all the factors that lie outside the individual that make the behaviour possible or prompt it. The single-headed and double-headed arrows in Figure 1 represent potential influence between components in the system. For example, opportunity can influence motivation as can capability; enacting a behaviour can alter capability, motivation, and opportunity.

A given intervention might change one or more components in the behaviour system. The causal links within the system can work to reduce or amplify the effect of particular interventions by leading to changes elsewhere. While this is a model of behaviour, it also provides a basis for designing interventions aimed at behaviour change. Applying this to intervention design, the task would be to consider what the behavioural target would be, and what components of the behaviour system would need to be changed to achieve that.

This system places no priority on an individual, group, or environmental perspective -- intra-psychic and external factors all have equal status in controlling behaviour. However, for a given behaviour in a given context it provides a way of identifying how far changing particular components or combinations of components could effect the required transformation. For example, with one behavioural target the only barrier might be capability, while for another it may be enough to provide or restrict opportunities, while for yet another changes to capability, motivation, and opportunity may be required.

Within the three components that generate behaviour, it is possible to develop further subdivisions that capture important distinctions noted in the research literature. Thus, with regard to capability, we distinguished between physical and psychological capability (psychological capability being the capacity to engage in the necessary thought processes - comprehension, reasoning *et al*.). With opportunity, we distinguished between physical opportunity afforded by the environment and social opportunity afforded by the cultural milieu that dictates the way that we think about things (*e.g*., the words and concepts that make up our language). With regard to motivation, we distinguished between reflective processes (involving evaluations and plans) and automatic processes (involving emotions and impulses that arise from associative learning and/or innate dispositions) [7,18,20]. Thus, we identified six components within the behavioural system (Figure 1). All, apart from reflective motivation, are necessary for a given behaviour but it is possible to generate a profile of which should be targeted to achieve the behavioural target.

### Systematic literature review of current frameworks

We used the following search terms to identify scholarly articles containing frameworks of behaviour change interventions: Topic = (taxonomy or framework or classification) AND Topic = ('behaviour change' or 'behavior change') AND Topic = (prevention OR intervention OR promotion OR treatment OR program OR programme OR policy OR law OR politics OR regulation OR government OR institute OR legislation).

Searches of Web of Science (Science and Social Science databases), Pubmed. and PsycInfo were supplemented by consulting with eight international experts in behaviour change, drawn from the disciplines of psychology, health promotion, epidemiology, public health, and anthropology. Given that there may be frameworks described in books and non peer-reviewed articles, we acknowledged that it was unlikely that we would arrive at a complete set, but we sought to canvass enough to be able to undertake an analysis of how well as a whole they matched the criteria described earlier and to achieve sufficient coverage of the key concepts and labels.

Documents were included if: they described a framework of behaviour change interventions (not specific behaviour change techniques); the framework was specified in enough detail to allow their key features to be discerned; and they were written in English. They were originally selected on the basis of titles and abstracts. A subset was then selected using the inclusion criteria for full review. The nature of the topic meant that this review could not be undertaken using the PRISMA guidelines [21].

Once the frameworks were identified, their categories and category definitions were extracted and tabulated. This was done independently by MS and a researcher who was not part of the study team or familiar with this work. The frameworks were coded according to the criteria for usefulness by RW and SM.

### Develop a new framework

The new framework was developed by tabulating the full set of intervention categories that had been identified and establishing links between intervention characteristics and components of the COM-B system that may need to be changed. The definitions and conceptualisation of the intervention categories were refined through discussion and by consulting the American Psychological Association's Dictionary of Psychology and the Oxford English Dictionary. The resulting framework was then compared with the existing ones in terms of the criteria of usefulness (*i.e*., met or not met).

Finally, a structure for the framework, in terms of organisation of components and links between them was arrived at through an iterative process of discussion and testing against specific examples and counter-examples. Linking interventions to components of the behaviour system was achieved with the help of a broad theory of motivation that encapsulated both reflective and automatic aspects, and focused on the moment to moment control of behaviour by the internal and external environment which in turn is influenced by that behaviour and the processes leading up to it [7]. Thus, for example, interventions that involved coercion could influence reflective motivation by changing conscious evaluations of the options or by establishing automatic associations between anticipation of the behaviour and negative feelings in the presence of particular cues. There is not the space to go into details of this analysis here. These can be found in [7].

### Test the reliability of the framework

The framework was used independently by RW and SM to classify the 24 components of the 2010 English government tobacco control strategy [22] and the 21 components of the 2006 NICE obesity guidance [23]. The level of inter-rater agreement was computed and any differences resolved through discussion. The areas of tobacco control and obesity reduction were chosen because these are among the most important in public health and ones where health professional behaviour has consistently been found to fall short of that recommended by evidence-based guidelines [24-26]. In addition, these documents cover a wide spectrum of behaviour change approaches. Following reliability testing and discussion of any disagreements, a 'gold standard' was established.

Next, reliability of use by practitioners was assessed by asking two policy experts (the Department of Health Policy Lead for implementation of the 2010 English government tobacco control strategy and a tobacco researcher) to independently classify the 24 components of the strategy (see Additional file 1 for coding materials). Their coding data were compared with the 'gold standard.'

## Results

### Systematic literature review of existing frameworks

From the systematic literature search, 1,267 articles were identified from the electronic databases, eight of which met our inclusion criteria. The expert consultations produced a further 17 articles, 11 of which met the inclusion criteria resulting in a total of 19 articles describing 19 frameworks. (See Additional file 2 for more detail of flow of studies through the review process, and Additional file 3 for reasons for exclusion). Additional file 4 shows the frameworks and gives a brief description of each [11,12,16,27-42].

Several things became apparent when reviewing the frameworks. First of all, it was clear it would be necessary to define terms describing categories of intervention more precisely than is done in everyday language in order to achieve coherence. For example, in everyday language 'education' can include 'training,' but for our purposes it was necessary to distinguish between 'education' and 'training' with the former focusing on imparting knowledge and developing understanding and the latter focusing on development of skills. Similarly we had to differentiate 'training' from 'modelling.' In common parlance, modelling could be a method used in training, but we use the term more specifically to refer to using our propensity to imitate as a motivational device. A third example is the use of the term 'enablement.' In everyday use, this could include most of the other intervention categories, but here refers to forms of enablement that are either more encompassing (as in, for example, 'behavioural support' for smoking cessation) or work through other mechanisms (as in, for example, pharmacological interventions to aid smoking cessation or surgery to enable control of calorie intake). There is not a term in the English language to describe that we intend, so rather than invent a new term we have stayed with 'enablement.'

Second, it became apparent that a distinction needed to be made between interventions (activities aimed at changing behaviour) and policies (actions on the part of responsible authorities that enable or support interventions). For example, an intervention that involved incentivising primary care organisations to prioritise public health interventions could be implemented through different policies such as producing guidelines and/or legislation. A second example is that raising the financial cost of a behaviour whose incidence one wishes to reduce (an example of coercion) could be enabled and supported by different policies, from fiscal measures (taxation) to legislation (fines). We therefore had to divide the categories that emerged into 'interventions' and 'policies.'

Third, any given intervention could in principle perform more than one behaviour change function. Thus the intervention categories identified from the 19 existing frameworks were better conceived of as non-overlapping functions: a given intervention may involve more than one of these. For example, a specific instance of brief physician advice to reduce alcohol consumption may involve the three different functions of education, persuasion, and enablement, whereas another may involve only one or two of these. With regard to the policies, it was possible to treat them as non-overlapping categories.

With this in mind, scrutiny of the frameworks yielded a set of nine intervention functions and seven policy categories that were included in at least one framework. Table 1 lists these and their definitions (their sources are detailed in Additional file 5). Additional file 6 shows whether or not the intervention functions and policy categories were covered by each of the reviewed frameworks. The inter-rater reliability for coding the frameworks by intervention functions and policy categories was 88%.

**Table 1:** Definitions of interventions and policies

Interventions	Definition	Examples
Education	Increasing knowledge or understanding	Providing information to promote healthy eating

Persuasion	Using communication to induce positive or negative feelings or stimulate action	Using imagery to motivate increases in physical activity

Incentivisation	Creating expectation of reward	Using prize draws to induce attempts to stop smoking

Coercion	Creating expectation of punishment or cost	Raising the financial cost to reduce excessive alcohol consumption

Training	Imparting skills	Advanced driver training to increase safe driving

Restriction	Using rules to reduce the opportunity to engage in the target behaviour (or to increase the target behaviour by reducing the opportunity to engage in competing behaviours)	Prohibiting sales of solvents to people under 18 to reduce use for intoxication

Environmental restructuring	Changing the physical or social context	Providing on-screen prompts for GPs to ask about smoking behaviour

Modelling	Providing an example for people to aspire to or imitate	Using TV drama scenes involving safe-sex practices to increase condom use

Enablement	Increasing means/reducing barriers to increase capability or opportunity^1^	Behavioural support for smoking cessation, medication for cognitive deficits, surgery to reduce obesity, prostheses to promote physical activity

**Policies**		

Communication/marketing	Using print, electronic, telephonic or broadcast media	Conducting mass media campaigns

Guidelines	Creating documents that recommend or mandate practice. This includes all changes to service provision	Producing and disseminating treatment protocols

Fiscal	Using the tax system to reduce or increase the financial cost	Increasing duty or increasing anti-smuggling activities

Regulation	Establishing rules or principles of behaviour or practice	Establishing voluntary agreements on advertising

Legislation	Making or changing laws	Prohibiting sale or use

Environmental/social planning	Designing and/or controlling the physical or social environment	Using town planning

Service provision	Delivering a service	Establishing support services in workplaces, communities etc.

**^1 ^**Capability beyond education and training; opportunity beyond environmental restructuring

Additional file 7 shows how existing frameworks met the criteria of usefulness. It is apparent that no framework covered all the functions and categories and thus did not meet the criterion of comprehensiveness. Only three frameworks met the criterion of coherence. Seven were explicitly linked to an overarching model of behaviour.

### Development of a new framework

**Figure 2:**
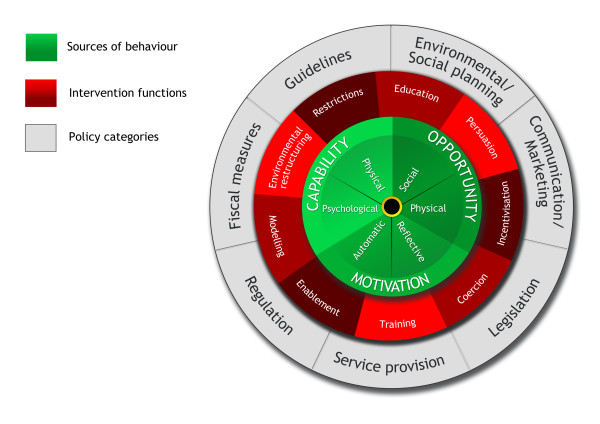
**The Behaviour Change Wheel**.

Given that policies can only influence behaviour through the interventions that they enable or support, it seemed appropriate to place interventions between these and behaviour. The most parsimonious way of doing this seemed to be to represent the whole classification system in terms of a 'behaviour change wheel' (BCW) with three layers as shown in Figure 2. This is not a linear model in that components within the behaviour system interact with each other as do the functions within the intervention layer and the categories within the policy layer.

Having established the structure of the new framework, the next step was to link the components of the behaviour system to the intervention functions and to link these to policy categories using the approach described in the Methods section. This led to a framework that met the third criterion of linkage with an overarching model of behaviour change (Tables 2 and 3).

**Table 2:** Links between the components of the 'COM-B' model of behaviour and the intervention functions

Model of behaviour: sources	Educa-tion	Persua-sion	Incentiv-isation	Coercion	Training	Restric-tion	Environ-mental restructuring	Model-ling	Enable-ment
C-Ph					√				√

C-Ps	√				√				√

M-Re	√	√	√	√					

M-Au		√	√	√			√	√	√

O-Ph						√	√		√

O-So						√	√		√

1. Physical capability can be achieved through physical skill development which is the focus of training or potentially through enabling interventions such as medication, surgery or prostheses

2. Psychological capability can be achieved through imparting knowledge or understanding, training emotional, cognitive and/or behavioural skills or through enabling interventions such as medication

3. Reflective motivation can be achieved through increasing knowledge and understanding, eliciting positive (or negative) feelings about behavioural target

4. Automatic motivation can be achieved through associative learning that elicit positive (or negative) feelings and impulses and counter-impulses relating to the behavioural target, imitative learning, habit formation or direct influences on automatic motivational processes (*e.g*., via medication)

5. Physical and social opportunity can be achieved through environmental change

**Table 3:** Links between policy categories and intervention functions

	Educat-ion	Persuas-ion	Incent-ivisation	Coerc-ion	Training	Restrict-ion	Environ-mental restructuring	Model-ling	Enable-ment
Communication/Marketing	√	√	√	√				√	

Guidelines	√	√	√	√	√	√	√		√

Fiscal			√	√	√		√		√

Regulation	√	√	√	√	√	√	√		√

Legislation	√	√	√	√	√	√	√		√

Environmental/social planning							√		√

Service Provision	√	√	√	√	√			√	√

### Testing the reliability of the new framework

The initial coding of the intervention functions and policy categories of the 2010 English Tobacco Control Strategy was achieved with an inter-rater agreement of 88%. The inter-rater agreement for the NICE Obesity Guidance was 79%. Differences were readily resolved through discussion (see Additional file 8 for details of the analysis). The percentage agreement between the identified components and the 'gold standard' was 85% for the implementation lead for the 2010 English government tobacco control strategy in the Department of Health and 75% for the tobacco researcher.

## Discussion

Within 19 frameworks for classifying behaviour change interventions, nine intervention functions and seven policy categories could be discerned. None of the frameworks covered all of these. Only a minority of the frameworks could be regarded as coherent or linked to an overarching model of behaviour. However, it was possible to construct a new BCW framework that did meet these criteria from the existing ones. This framework could be reliably applied to classify interventions within two important areas of public health.

We believe that this is the first attempt to undertake a systematic analysis of behaviour intervention frameworks and apply usefulness criteria to them. This is also the first time that a new framework has been constructed from existing frameworks explicitly to overcome their limitations. Moreover, we are not aware of other attempts to assess the reliability with which a framework can be applied in practice.

It must be recognised that there are a near infinite number of ways of classifying interventions and intervention functions. The one arrived at here will no doubt be superseded. But for the present, it has the benefits of having been derived from classifications already available and therefore covering concepts that have previously been considered to be important, and using an overarching model of behaviour to link interventions to potential behavioural targets. The most important test of this framework will be whether it provides a more efficient method of choosing the kinds of intervention that are likely to be appropriate for a given behavioural target in a given context and a given population.

Just by identifying all the potential intervention functions and policy categories this framework could prevent policy makers and intervention designers from neglecting important options. For example, it has been used in UK parliamentary circles to demonstrate to Members of Parliament that the current UK Government is ignoring important evidence-based interventions to change behaviour in relation to public health [43,44]. By focusing on environmental restructuring, some incentivisation and forms of subtle persuasion to influence behaviour, as advocated by the popular book 'Nudge' [45], the UK Government eschews the use of coercion, persuasion, or the other BCW intervention functions that one might use.

Although awareness of the full range of interventions and policies is important for intervention design, the BCW goes beyond providing this. It forms the basis for a systematic analysis of how to make the selection of interventions and policies (as in Tables 2 and 3). Having selected the intervention function or functions most likely to be effective in changing a particular target behaviour, these can then be linked to more fine-grained specific behaviour change techniques (BCTs). Any one intervention function is likely to comprise many individual BCTs, and the same BCT may serve different intervention functions. An examination of BCTs used in self-management approaches to increasing physical activity and healthy eating [46], and in behavioural support for smoking cessation [47,48], shows that these BCTs serve five of the intervention functions: education, persuasion, incentivisation, training, and enablement. The other four intervention functions (coercion, restriction, environmental restructuring, and modelling) place more emphasis on external influences and less on personal agency. Reliable taxonomies for BCTs within these intervention functions have yet to be developed.

One of the strengths of this framework is that it incorporates context very naturally. There is a general recognition that context is key to the effective design and implementation of interventions, but it remains under-theorised and under-investigated. The 'opportunity' component of the behavioural model is the context, so that behaviour can only be understood in relation to context. Behaviour in context is thus the starting point of intervention design. The behaviour system also has automatic processing at its heart, broadening the understanding of behaviour beyond the more reflective, systematic cognitive processes that have been the focus of much behavioural research in implementation science and health psychology (for example, social cognition models such as the Theory of Planned Behaviour).

An existing framework that has made an important contribution to making intervention design more systematic is 'intervention mapping' [16]. A key difference between this and the BCW approach is that intervention mapping aims to map behaviour on to its 'theoretical determinants' in order to identify potential levers for change, whereas the BCW approach recognises that the target behaviour can in principle arise from combinations of any of the components of the behaviour system. It may appear that some components are more important than others because of a lack of variance in (including absence or universal presence of) the variables concerned in the population under study. This can be illustrated by a study of GP advice to smokers, which found that a single variable -- degree of concern that it would harm the doctor-patient relationship -- accounted for significant variance in the rate of advice-giving [49]. 'Intervention mapping' would suggest that concern be the target for an intervention (as long as a judgement were made that this could be modified using interventions that were realistically applicable). The BCW would analyse the target behaviour in context and note that, regardless of what covariation might currently exist, the target behaviour consists of an activity in which capability is not at issue, and the reflective motivation is broadly positive. The problem arises because automatic motivational factors are currently working against the behaviour (*e.g*., lack of emotional reward for giving advice or punishment for not giving it and lack of cues to action). Moreover, the physical opportunity is limited (lack of time) and the social opportunities are also somewhat limited. It would then consider the full range of ways in which the frequency of advice-giving could be increased. Because the target behaviour is part of a 'system,' a single intervention may have consequences for other parts of the system - these might work against sustainable change or in favour of it.

Thus, the BCW approach is based on a comprehensive causal analysis of behaviour and starts with the question: 'What conditions internal to individuals and in their social and physical environment need to be in place for a specified behavioural target to be achieved?' The 'intervention mapping' approach is based on an epidemiological analysis of co-variation within the behavioural domain and starts with the question: 'What factors in the present population at the present time underlie variation in the behavioural parameter?' When it comes to theoretical underpinnings, the BCW approach draws from a single unifying theory of motivation in context that predicts what aspects of the motivational system will need to be influenced in what ways to achieve a behavioural target, whereas the 'intervention mapping' approach draws on a range of theoretical approaches each of which independently addresses different aspects of the behaviour in question.

The BCW is being developed into a theory- and evidence-based tool allowing a range of users to design and select interventions and policies according to an analysis of the nature of the behaviour, the mechanisms that need to be changed in order to bring about behaviour change, and the interventions and policies required to change those mechanisms. An ongoing programme of research is developing an 'intervention design tool' based on the BCW. It starts with a theoretical understanding of behaviour to determine what needs to change in order for the behavioural target to be achieved, and what intervention functions are likely to be effective to bring about that change. It is being field tested by a range of staff involved in policy and intervention work applying the framework to develop prototype strategies for specific implementation targets. Data are being collected about ease of use and the potential of the BCW to generate new insights.

There are a number of limitations to the research described in this paper. First, it is possible that the systematic review missed important frameworks and/or intervention functions. Second, judgement is inevitably involved in conceptualising intervention functions and policy categories. There are many different ways of doing this, and no guarantees that the one arrived at here is optimal. Indeed, different frameworks may be more or less useful in different circumstances. Third, even though the proposed framework appears to be comprehensive and can be used reliably to characterise interventions, it is possible that it may prove difficult to use. However, the systematic way in which development of the BCW has been approached should enable it to provide a more robust starting point for development of improved frameworks than has hitherto been possible.

## Competing interests

The authors declare that they have no competing interests.

## Authors' contributions

SM and RW conceived the study, designed the measures, supervised the systematic review, supervised the analyses and drafted the write-up. MMvS undertook the systematic review, performed the coding and commented on the write-up. All authors read and approved the final manuscript.

## Supplementary Material

Additional file 1 **Applying the Behaviour Change Wheel to characterise intervention strategies: Coding Materials**. Behaviour Change Wheel Coding materials Click here for file

Additional file 2 **Flow of studies through the review process**. Flow of studies through the review process Click here for file

Additional file 3 **Reports excluded from the review**. Reports excluded from the review Click here for file

Additional file 4 **Intervention frameworks**. Analysis of intervention frameworks Click here for file

Additional file 5 **Sources of definitions of interventions and policies**. Sources of definitions of interventions and policies Click here for file

Additional file 6 **How existing frameworks map on to intervention and policy categories**. How existing frameworks map on to intervention and policy categories Click here for file

Additional file 7 **Frameworks analysed by criteria of comprehensive coverage, coherence and link to a model of behaviour**. Analysis by criteria of comprehensive coverage, coherence and link to a model of behaviour Click here for file

Additional file 8 **BCW classification of the English 2010 Tobacco Control Strategy and the NICE Obesity Guidelines (2006)**. BCW classification of the English 2010 Tobacco Control Strategy and the NICE Obesity Guidelines (2006) Click here for file
