# Supplementary material for: The behaviour change wheel: A new method for characterising and designing behaviour change interventions
Source: Implement Sci. 2011 Apr 23;6:42. doi: 10.1186/1748-5908-6-42 (PMC3096582; doi:10.1186/1748-5908-6-42)
Supplement: Additional file 1 — Applying the Behaviour Change Wheel to characterise intervention strategies: Coding Materials. Behaviour Change Wheel Coding materials [file 1748-5908-6-42-S1.PDF]

Additional file 1

Applying the Behaviour Change Wheel to characterise intervention strategies: Coding Materials

1. Familiarise yourself with the definitions of the Intervention and Policy categories. See Table below.

2. Establish the target of the intervention strategy (whose behaviour is being changed)

3. For each intervention and policy, establish the agent of change (who is enacting it).

4. Statements of general exhortation that do not specify an Intervention or Policy should be coded as U.

5. Code the Intervention first, followed by the Policy. Identify which Policies are (a) specified and (b) would be needed in order to enact the Interventions.

    a. Example: the strategy ‘Remove tobacco products from display in shops’ could only be enacted by the Policy of Legislation (even thought this is not stated)
    b. If it is unclear what policy is being proposed or if many could be used, code U.

6. Do not infer beyond what is directly implied.

    a. Example: a strategy which involves ‘Encourage’ would definitely involve ‘Persuasion’, but may not involve ‘Education’.

7. Where a change or improvement is proposed to an existing Intervention or Policy, code for the original Intervention or Policy.

    a. Example: If an intervention strategy involves putting more resources into a given service provision to enable a behaviour, code as ‘Enablement’ and ‘Service Provision’.
