# Supplementary figures and images for: The behaviour change wheel: A new method for characterising and designing behaviour change interventions
Source: Implement Sci. 2011 Apr 23;6:42. doi: 10.1186/1748-5908-6-42 (PMC3096582; doi:10.1186/1748-5908-6-42)

## Additional file 2

### Flow of studies through the review process

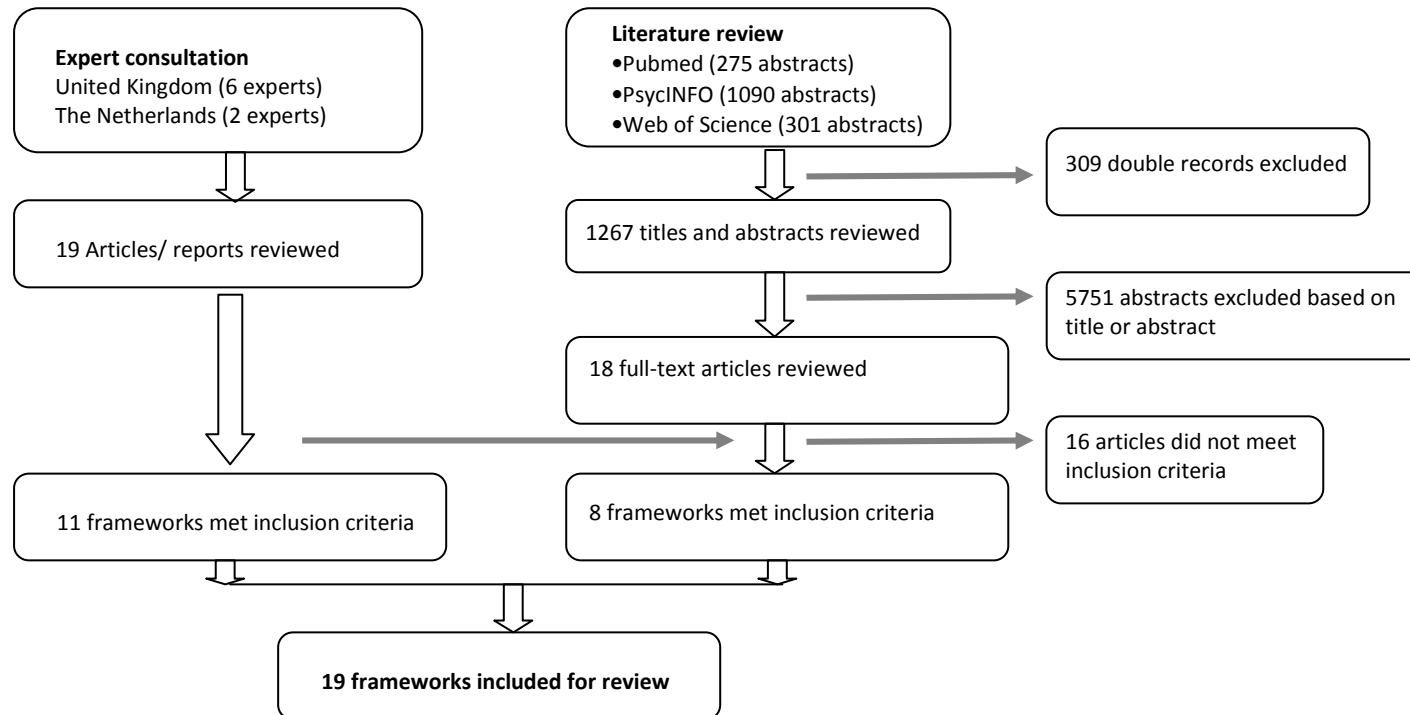

Supplement: Additional file 2 — Flow of studies through the review process. Flow of studies through the review process [file 1748-5908-6-42-S2.PDF]
