# Supplementary material for: The behaviour change wheel: A new method for characterising and designing behaviour change interventions
Source: Implement Sci. 2011 Apr 23;6:42. doi: 10.1186/1748-5908-6-42 (PMC3096582; doi:10.1186/1748-5908-6-42)
Supplement: Additional file 3 — Reports excluded from the review. Reports excluded from the review [file 1748-5908-6-42-S3.PDF]

Additional file 3

Reports excluded from the review

**Framework**	**Author**	**Reason excluded**
Personal responsibility and changing behaviour	Prime Minister's strategy unit (2004)	Not a taxonomy. However it describes possible behaviour change interventions
Gezond Gedrag Bevorderd (promoting health behaviour)(2)	IBO prevention (2007)	This is not a framework, just a description of the current policy in the Netherlands. However two striking categories mentioned were:<br>1. Providing information (i.e. mass media campaigns, product labelling, education programs)<br>2. covenant/ agreements with the industry, municipalities etc
European WHO Second Action Plan for Food and Nutrition Policy(3)	European WHO (2008)	This is not a framework, but an action plan that aims to improve the health of the general population with regard to nutrition and nutritional safety, notably by providing comprehensive information and education to consumers (including developing guidelines, conduct public (mass media) campaigns, ensure appropriate marketing practices, promote adequate food labelling).
PRECEDE-PROCEED framework(4)	Green and Kreuter (2005)	Intervention planning model, no intervention techniques are described. Provides a framework for assessing health and quality-of-life needs and for designing, implementing, and evaluating health promotion and other public health programs to meet those needs.
RE-AIM framework(5)	Glasgow, Vogt, & Boles (1999)	Does not refer to specific intervention or policy techniques aimed at changing behaviour. Planning models aimed at encouraging intervention developers to pay more attention to essential program elements that can improve the sustainable adoption and implementation of effective, generalizable, evidence-based interventions The five steps to translate research into action are:<br>1. Reach the target population<br>2. Effectiveness or efficacy<br>3. Adoption by target settings or institutions<br>4. Implementation, consistency of delivery of intervention<br>5. Maintenance of intervention effects in individuals and settings over time
Nine Principles Model(6)	Government Social Research (2008)	Intervention planning model: does not provide intervention/ policy techniques. Aims to integrate behavioural models with theoretical understanding of effective approaches to change. Provides a starting point for selecting methods and developing behaviour change intervention. The nine principles are:<br>1. Identify the audience groups and the target behaviour<br>2. Identify relevant behavioural models<br>3. Selecting the key influencing factors<br>4. Identify effective intervention techniques<br>5. Engage the target audience for the intervention

		6. Develop a prototype intervention<br>7. Pilot the intervention<br>8. Evaluate impacts and processes<br>9. Feedback learning from the evaluation.
Commissioning and Behaviour Change; kicking bad habits(7)	The King's fund (2008)	Not a taxonomy. However it describes which methods are currently used in changing several health behaviours:<br>1. reducing exposure<br>2. reducing the availability and supply of (tobacco) products<br>3. effectively regulating (tobacco) products<br>*4.* running effective communications and education campaigns<br>*5.* reducing advertising, marketing and promotion restrict the availability of tobacco products by regulating supply, reducing tobacco advertisement and promotion, and regulating labelling and packaging
Framework for Health promotion and Disease prevention programs(8)	Winett (1995)	Planning model on the systematic development of a health promotion or disease prevention intervention, and describes 6 basic sections<br>1. Epidemiological and health indicators i<br>2. Goals for nation<br>3. Methods (e.g. health promotion, protection)<br>4. Approach (timing (primary, secondary, tertiary prevention), level (e.g. individual, group)<br>5. Marketing plan<br>6. Intervention design, implementation and evaluation
Events of instruction framework (modified) (9)	Kinzie (2005)	Taxonomy of five instructional design strategies for health education interventions based on the conditions of learning framework, describing lower level behaviour change techniques:<br>1. Gain attention (e.g. convey health threats and benefits)<br>2. Present stimulus material (e.g. tailor message to audience knowledge and values)<br>3. provide learning guidance (e.g. use trustworthy models to demonstrate)<br>4. elicit performance and provide feedback<br>5. enhance retention and transfer (e.g. provide social supports and deliver behavioural cues).
Social marketing framework(10)	Morris and Clarkson (2009)	Description of how social marketing can be used as a generic framework for analysing barriers to the take-up of clinical guidelines, and planning interventions which seek to enable this change. Does not provide a framework of intervention/ policy strategies
Framework to guide intervention design and implementation (11)	Poland, Krupa and McCall (2009)	Planning model, does not provide an overview of intervention/ policy strategies. Takes a "settings approach" to health promotion(e.g. addressing the contexts within which people live, work, and play) and makes these the object of inquiry and intervention increase the likelihood of success. Comprises three parts: (a) understanding settings, (b) changing settings, and (c) knowledge development and knowledge translation
Healthy impact	Frieden (2010)	A 5-tier pyramid that describes the impact of different

pyramid(12)		types of public health interventions and provides a framework to improve health (1) efforts to address socioeconomic determinants of health interventions, (greatest potential impact) (2) interventions that change the context to make individuals' default decisions healthy, (3) clinical interventions that require limited contact but confer long-term protection, (4) direct clinical care, and (5) health education and counselling. No intervention strategies/ policies are described.
Actor-network Theory(13)	Young, Borland and Coghill (2010)	Does not describe a framework of intervention strategies/ policies. This study examined the key attributes of smoke-free regulations and policy innovations that were successfully based on the actor-network theory.
Complex Adaptive System Framework(14)	Leykum, Parchman, Pugh. Lawrence, Noel and McDaniel (2010)	The framework distinguishes characteristics of clinical systems in which patients receive care. The clinical systems are defined as "environments in which individuals learn, inter-relate, self-organize and co-evolve in response to changes in their internal and external environments". Focuses on characteristics of individuals and not of interventions: individuals' capability to learn; the interconnections between individuals; the ability of participants to self-organize and the tendency of participants to co-evolve.
Framework of exposure to internet-delivered intervention(15)	Crutzen, de Nooijer, Brouwer, Oenema, Brug and de Vries (2009)	The framework incorporates elements of user experiences of websites and can be used for intervention developers in order to increase the exposure rates of internet-delivered interventions. The framework does not include intervention characteristics aimed at changing behaviour.
Framework for cardiovascular disease prevention approaches(16)	Schmid, Pratt and Howze (1995)	The paper describes the approaches used to change environment, policy and individual behaviour in order to prevent cardiovascular disease. No framework describing intervention characteristics or policies is described.

Reference List

(1) Prime Minister's Strategy Unit, the Cabinet Office. Personal Responsibility and Changing Behaviour: the state of knowledge and its implications for public policy. London: Strategy Unit; 2004.

(2) Interdepartementaal beleidsonderzoek (IBO). Gezond gedrag bevorder (*promoting health beaviour*). 2007.

(3) WHO Europe. WHO European Action Plan for Food and Nutrition Policy 2007-2012. Copenhagen: World Health Organization; 2008.

(4) Green L, Kreuter M. Health program planning: an educational and ecological approach. 4 ed. New York: McGraw- Hill; 2005.

(5) Glasgow RE, Vogt TM, Boles SM. Evaluating the public health impact of health promotion interventions: the RE-AIM framework. American Journal of Public Health 1999 Sep;89(9):1322-7.

(6) Government Social Research Unit. GSR behaviour change practice guide. London: Government Social Research Unit; 2008. Report No.: PU547.

(7) Boyce T, Robertson R, Dixon A. Commisioning and Behaviour Change; Kicking Bad Habits final report. London: the King's Fund; 2008.

(8) Winett RA. A framework for health promotion and disease prevention programs. American Psychologist 1995 May;50(5):341-50.

(9) Kinzie MB. Instructional design strategies for health behavior change. Patient Educ Couns 2005 Jan;56(1):3-15.

(10) Morris ZS, Clarkson PJ. Does social marketing provide a framework for changing healthcare practice? Health Policy 2009 Jul;91(2):135-41.

(11) Poland B, Krupa G, McCall D. Settings for health promotion: an analytic framework to guide intervention design and implementation. Health Promot Pract 2009 Oct;10(4):505-16.

(12) Frieden TR. A framework for public health action: the health impact pyramid. Am J Public Health 2010 Apr;100(4):590-5.

(13) Young D, Borland R, Coghill K. An actor-network theory analysis of policy innovation for smoke-free places: understanding change in complex systems. Am J Public Health 2010 Jul;100(7):1208-17.

(14) Leykum LK, Parchman M, Pugh J, Lawrence V, Noel PH, McDaniel RR, Jr. The importance of organizational characteristics for improving outcomes in patients with chronic disease: a systematic review of congestive heart failure. Implement Sci 2010;5:66.

(15) Crutzen R, de NJ, Brouwer W, Oenema A, Brug J, de Vries NK. A conceptual framework for understanding and improving adolescents' exposure to Internet-delivered interventions. Health Promot Int 2009 Sep;24(3):277-84.

(16) Schmid TL, Pratt M, Howze E. Policy as intervention: environmental and policy approaches to the prevention of cardiovascular disease. Am J Public Health 1995 Sep;85(9):1207-11.
