# Supplementary material for: The behaviour change wheel: A new method for characterising and designing behaviour change interventions
Source: Implement Sci. 2011 Apr 23;6:42. doi: 10.1186/1748-5908-6-42 (PMC3096582; doi:10.1186/1748-5908-6-42)
Supplement: Additional file 4 — Intervention frameworks. Analysis of intervention frameworks [file 1748-5908-6-42-S4.DOC]

Additional file 4

Intervention frameworks 1 to 9

|  | **1. Epicure taxonomy [11]** | **2. Culture capital framework [12]** | **3. EPOC taxonomy of interventions [16]** | **4. RURU: Intervention implementation taxonomy [27]** | **5. MINDSPACE [28]** | **6. Taxonomy of behaviour change techniques [29]** | **7. Intervention mapping [30]** | **8. People and places framework [31]** | **9. Public health: ethical issues [32]** |
| --- | --- | --- | --- | --- | --- | --- | --- | --- | --- |
| **Author** | West (2006) | Knott *et al.* (2008) | Cochrane Effective Practice and Organisation of Care  Review Group (EPOC) (2010) | Walter *et al.* (2003) | Institute for Government and Cabinet Office (2010) | Abraham *et al.* (2010) | Bartholomew *et al.* (2011) | Maibach *et al.* (2007) | Nuffield Council on Bioethics (2007) |
| **Description** | Taxonomy of approaches designed to influence behaviour patterns | Framework of knowledge about culture change, offering practical tools for policymaking | Checklist to guide systematic literature reviewers about the types of information to extract from primary studies | Taxonomy covering a wide range of policy, practice and organisational targets aimed at increasing impact of research | Checklist for policy-makers aimed at changing or shaping behaviour | Taxonomy of behaviour change techniques grouped by change targets | Protocol for a systematic development of theory- and evidence-based interventions | Framework that explains how communication and marketing can be used to advance public health | Ladder of interventions by government, industry, organisations and individuals to promote public health. |
| **Details** | Approaches aimed at reducing the number of people who use tobacco by preventing young people form starting or motivating smokers to stop:   1. Educate 2. Persuade 3. Incentivise 4. Coerce 5. Upskill 6. Regulate 7. Empower | Practical tools for policymaking, including:  1. Support through most immediate influences (*e.g.*, interventions that promote parenting, peers, and role models)  2. Support through wider social influences (*e.g.*, role of national dialogue or government leading by example)  3. Enabling through providing capacity and alternatives for different choices  4. Encouraging through incentives or use of legislation and regulation | Four main categories:  1. Professional interventions (*e.g.*, educational materials, meetings)  2. Financial interventions (*e.g.*, incentives, penalties)  3. Organisational interventions (*e.g.*, changes in setting, structure or systems)  4. Regulatory interventions (*e.g.*, changes in medical liability). The first three categories are, if applicable, subdivided into provider oriented, patient oriented or structural interventions. | Categories of *intervention type* according to form and content) (*e.g.*, seminars, conferences, partnerships) and *intervention mechanisms* according to theoretical framework (*e.g.*, dissemination, education, social influence) | Nine ways policy-makers can use to influence behaviour:  1. Messenger  2. Incentives  3. Norms  4. Defaults  5. Salience  6. Priming  7. Affect  8. Commitments  9. Ego | Behaviour change techniques designed to change:  1. cognitive and affective attitudes  2. risk perception  3. normative beliefs  4. identity  5. goal setting and motivation  6. self-efficacy and self-regulation  7. encouragement and rewards  8. environmental prompts or reminders from others  9. emotional states in readiness for action  (10) social skills | Theoretical methods and practical strategies for intervention design, targeting  1. knowledge  2. awareness  3. risk perception  4. habit  5. attitude  6. social influence  7. skills/ self-efficacy  8. environment  9. social norms  10. social networks | Influence of communication (*i.e.,* health communication on the individual/ people level and policy advocacy on the places level) and marketing (*i.e.,* social marketing on the people level and organizational marketing on the places level), with each having potential to influence behaviour in five fields:  1. individuals  2. social networks  3. population  4. local-level places  5. distal level places | Ways that public health policies can affect people’s choices, whereby interventions higher up the ladder (*e.g.*, eliminate choice, restrict choice) are more intrusive and therefore require a stronger justification than intervention lower down the ladder (*e.g.*, enable choice, provide information, monitor current situation). |

Intervention frameworks 10 to 19

|  | **10. Injury control framework [33]** | **11. Implement-ation taxonomy [34]** | **12. Legal framework [35]** | **13. PETeR [36]** | **14. DEFRA’s 4E model [37]** | **15. STD/ HIV framework [38]** | **16. Framework on public policy in physical activity [39]** | **17. Interven-tion framework for retail pharmacies [40]** | **18. Environ-mental policy framework [41]** | **19. Population Services International (PSI) framework [42]** |
| --- | --- | --- | --- | --- | --- | --- | --- | --- | --- | --- |
| **Author/ Institute** | Geller *et al.* (1990) | Leeman *et al.* (2007) | Perdue *et al.* (2005) | White (in prep.) | DEFRA (2008) | Cohen and Scribner (2000) | Dunton *et al.* (2010) | Goel *et al.* (1996) | Vlek (2000) | PSI (2004) |
| **Description** | Heuristic framework for categorising and evaluating behaviour change strategies aimed at controlling injuries | Theory-based taxonomy of methods for implementing change in practice | Conceptual framework for identifying possible legal strategies used for preventing cardiovascular diseases | Comprehensive and universally applicable model or taxonomy of health interventions. | Process model for policy makers aimed at promoting pro-environmental behaviours in accordance with social marketing principles | Taxonomy to expand the scope of interventions that can be used to prevent STD and HIV transmission | Taxonomy aimed at understanding how and why policies successfully impact on behaviour change | Framework that presents factors that may affect retail pharmacy describing and strategies for behaviour change to improve appropriateness of prescribing | A taxonomy of major environmental problems, their different levels and global spheres of impact, and conceptual modelling of environmental problem- solving | A conceptual framework to guide and help conduct research on social marketing interventions |
| **Details** | Twenty-four different approaches to change behaviour derived from a literature review, categorised into:  1. communication/ education approaches  2. activators and consequences. They were scored on their intervention effectiveness. | Fourteen implementation methods organised into five categories:  1. Increasing coordination  2. raising awareness  3. persuasion via interpersonal channels  4. persuasion via reinforcing belief that behaviour will lead to desirable results and (5) increasing behavioural control | Seven legal strategies  1. direct regulation  2. economic incentives and disincentives (3) indirect regulation through private enforcement  4. government as information provider  5. government as direct provider of services  6. government as employer and landlord  7. laws directed at other levels of government | Four main components:  1. Policy (*e.g.*, legal or fiscal regulations aimed at enhancing health)  2. Education (eg. learning experiences and communications that enable people to increase control over health)  3. Technology (eg, technical innovation that can be used to promote health)  4. Resources (eg. financial or material resources that facilitates or enables health) | Four dimensions:  1. Enable: core infrastructure (eg. remove barriers, give information, provide facilities)  2. Encourage: fiscal, legislative and regulatory measures (eg. tax system, reward scheme, expenditure)  3. Engage: communications (*e.g.*, community action, co-production)(4) Exemplify: government demonstrating its commitment to behaviour (eg. leading by example). | Two categories of interventions:  1. individual-level (*i.e.,* interventions that targets risk factors attributable to individuals, such as counselling, screening and treatment)  2. structural level (*i.e.,* interventions that target conditions outside the control of the individual such as addressing accessibility, social and physical structures) | Describes policy strategies aimed at modifying aspects of social, physical, economic and educational environments and how they map onto health behaviour theoretical variables and processes thought to lead to physical activity behaviour change | Four categories of interventions  1. information alone  2. persuasion  3. incentives  4. coercion. | Behavioural processes and theories and 7 strategies for social behaviour change:  1. Provision of physical alternatives, (re)arrangements:  2. Regulation-and-enforcement  3. Financial-economic stimulation  (4) Provision of information, education, communication  5. Social modeling and support  (6) Organizational change  (7) Changing values and morality | A framework of four levels of behavioral determinants and their relationship with health:  1. goal (health status and quality of life)  2. purpose (use, risk-reducing status and need)  3. outputs (opportunity, ability, motivation and population need)  4. activities (social marketing intervention, product, place, price, promotion) |
