# Supplementary material for: The behaviour change wheel: A new method for characterising and designing behaviour change interventions
Source: Implement Sci. 2011 Apr 23;6:42. doi: 10.1186/1748-5908-6-42 (PMC3096582; doi:10.1186/1748-5908-6-42)
Supplement: Additional file 5 — Sources of definitions of interventions and policies. Sources of definitions of interventions and policies [file 1748-5908-6-42-S5.PDF]

Additional file 5

Sources of definitions of interventions and policies

	**Label**	**APA**	**OED**
Interventions			
1.	Education	The process of teaching knowledge, skills and values	Systematic instruction or training
2.	Persuasion	Active attempt by one person to change another person's attitudes, beliefs or emotions associated with some issue, person, concept, or object	the addressing of arguments or appeals to a person in order to induce cooperation, submission, or agreement
3.	Incentivisation	Using an external stimulus, such as a condition or an object, that enhances or serves as a motive for behaviour	Arousing feeling, or inciting to action
4.	Coercion	The process of attempting to influence another person through the exercise of physical, psychological or social power	Constraint, restraint, compulsion; the application of force to control the action of a voluntary agent
5.	Training	Systematic instruction and practice by which an individual acquires competence in a specific discipline, talent, or vocational or recreational skills of activity	systematic instruction and exercise in some art, profession, or occupation, with a view to proficiency in it
6.	Restriction		Something that restricts a person or thing; a limitation on action
7.	Environmental restructuring		GOOGLE – restructuring- reorganizing or rearranging
8.	Modelling	A technique in which learning occurs through observation and imitation alone	The action or process of eliciting a mode of behaviour by example
9.	Enablement	Enablement: Encouraging or allowing people to meet their own needs and achieve desired ends	The process of rendering able, competent, or powerful/<br>source of help, information, strength, etc.
Policies			
1.	Communication/ marketing	Communication: The transmission of information, verbal or non-verbal	The transmission or exchange of information, knowledge, or ideas, by means of speech, writing, mechanical or electronic media, etc/<br>the action, business, or process of promoting and

			selling a product, etc., including market research, advertising, and distribution.
2.	Guidelines		GOOGLE - any document that aims to streamline particular processes according to a set routine.
3.	Fiscal		Of or pertaining to the fisc or treasury of a state or prince; pertaining to the public revenue.
4.	Regulation		A rule or principle governing behaviour or practice; *esp.* such a directive established and maintained by an authority.
5.	Legislation		The action of making or giving laws; the enactment of laws, lawgiving;
6.	Environmental/ social planning		Planning= the designing or controlling of urban or economic development
7.	Service provision		GOOGLE - delivery of the service directly by agency staff or authorization for the purchase of the service from another provider
