# Supplementary material for: The behaviour change wheel: A new method for characterising and designing behaviour change interventions
Source: Implement Sci. 2011 Apr 23;6:42. doi: 10.1186/1748-5908-6-42 (PMC3096582; doi:10.1186/1748-5908-6-42)
Supplement: Additional file 6 — How existing frameworks map on to intervention and policy categories. How existing frameworks map on to intervention and policy categories [file 1748-5908-6-42-S6.PDF]

Additional file 6

How existing frameworks map on to intervention and policy categories (1 to 9)

	1. Epicure Taxonomy	2. Culture Capital framework	3. EPOC taxonomy of interventions	4. RURU: Intervention implementation taxonomy	5. MINDSPACE	6. Taxonomy of Behaviour Change Techniques	7. Intervention Mapping	8. People and places framework	9. Public Health: ethical issues
**Intervention system**	√								
Education	√	√	√	√		√	√		
Persuasion	√		√		√	√	√		
Incentivisation	√	√	√	√	√	√	√		
Coercion	√	√		√	√		√		
Training	√					√	√		
Restriction	√								
Environmental restructuring		√	√		√	√	√		
Modelling		√					√		
Enablement/	√			√		√	√		
**Policy system**									
Communication/ Marketing		√	√				√	√	√
Guidelines		√							
Fiscal		√							√
Regulation		√	√				√		√
Legislation		√							√
Environmental/ social planning		√					√		
Service Provision		√	√				√		

How existing frameworks map on to intervention and policy categories (10 to 19)

	**10. Injury Control Framework**	**11. Implementation taxonomy**	**12. Legal Framework**	**13. PETeR**	**14. DEFRA's 4E model**	**15. STD/ HIV framework**	**16. Framework on Public Policy in Physical activity**	**17. Interven-tion framework for retail pharmacies**	**18. Environ-mental policy framework**	**19. PSI Framework**
**Intervention system**										
Education	√	√		√	√	√		√	√	√
Persuasion	√	√				√		√		√
Incentivisation	√			√	√		√	√		√
Coercion					√			√		√
Training				√	√	√				
Restriction										
Environmental restructuring	√					√	√		√	√
Modelling	√				√				√	
Enablement	√	√		√	√					
**Policy system**										
Communication/ Marketing			√	√		√	√			√
Guidelines	√			√					√	
Fiscal			√	√	√				√	√
Regulation			√	√		√	√		√	
Legislation			√	√						
Environmental/ social planning			√			√			√	√
Service Provision			√							
