# Supplementary material for: The behaviour change wheel: A new method for characterising and designing behaviour change interventions
Source: Implement Sci. 2011 Apr 23;6:42. doi: 10.1186/1748-5908-6-42 (PMC3096582; doi:10.1186/1748-5908-6-42)
Supplement: Additional file 7 — Frameworks analysed by criteria of comprehensive coverage, coherence and link to a model of behaviour. Analysis by criteria of comprehensive coverage, coherence and link to a model of behaviour [file 1748-5908-6-42-S7.PDF]

Additional file 7

Frameworks analysed by criteria of comprehensive coverage, coherence and link to a model of behaviour

	Epicure Taxonomy (1)	Culture Capital framework (2)	EPOC taxonomy of interventions(3)	RURU: Intervention implementation Taxonomy (4)	MINDSPACE (5)	Taxonomy of Behaviour Change Techniques(6)	Intervention Mapping (7)	People and places framework (8)	Public Health: ethical issues (9)	Injury Control Framework (10)	Implementation taxonomy (11)	Legal Framework(12)	PETeR (13)	Defra's 4E model (14)	STD/ HIV framework(15)	Framework on Public Policy in Physical activity(16)	Intervention framework for retail pharmacies(17)	Environmental policy framework(18)	PSI Framework (19)
**Comprehensiveness**																			
Framework covers all identified categories																			
**Coherence**																			
Framework does not mix type and specificity of entities					√	√		√											
**Model**																			
Framework is based on a model of behaviour or behaviour change						√	√				√		√	√		√			√
