# Supplementary material for: The behaviour change wheel: A new method for characterising and designing behaviour change interventions
Source: Implement Sci. 2011 Apr 23;6:42. doi: 10.1186/1748-5908-6-42 (PMC3096582; doi:10.1186/1748-5908-6-42)
Supplement: Additional file 8 — BCW classification of the English 2010 Tobacco Control Strategy and the NICE Obesity Guidelines (2006). BCW classification of the English 2010 Tobacco Control Strategy and the NICE Obesity Guidelines (2006) [file 1748-5908-6-42-S8.PDF]

Additional file 8

BCW classification of the English 2010 Tobacco Control Strategy and the NICE Obesity Guidelines (2006)

English 2010 Tobacco Control Strategy

Behaviour change target: Reducing smoking prevalence in England to 15% by 2020.

Context: A country with adult smoking prevalence of 21% (having declined by 25% over the preceding 10 years but currently showing no evidence of decline) at the end of a 10-year period of tobacco control activity involving high levels of tobacco taxation, substantial anti-smuggling enforcement, a ban on smoking in indoor public areas, a ban on promotion of tobacco products except at point-of-sale and on the internet, free NHS Stop Smoking Services, substantial spending on mass media campaigns, and graphic health warnings on cigarette packets.

Coding Key:
Interventions
Education E, Persuasion P, Incentivisation I, Coercion C, Training T, Restriction R, Environmental restructuring V, Modelling M, Enablement/resources N, Unclassifiable U
Policies
Fiscal F, Communication/marketing C, Service provision S, Legislation L, Regulation R, Guidelines G, Environmental/social planning E, Unclassifiable U

This Table: Score 2 if both coders correct for both intervention and policy, 1.5 if both coders agree for intervention or policy and overlap but do not completely agree for the other e.g. if one coder gives E,P while the other coder give E,N. Italicised sections did not specify interventions or policies.

	Intervention function	Policy category	Agreement (n)
**Reducing smoking uptake**			
1. Make tobacco less affordable by continuing to consider the case for real increases in duty on tobacco on a Budget-by-Budget basis, and by additional investment in overseas Fiscal Crime Liaison Officers, whom we expect to prevent over 200 million illicit cigarettes from being smuggled into the UK each year. More broadly we will continue to bear down on the market for illicit cigarettes, which has fallen from 21% in 2000 to 10% in 2007/08 (midpoint estimates), and achieve similar success in reducing the illicit market for hand-rolled tobacco.	C	F	2
2. Remove tobacco products from display in shops.	V	L	2
3. Prohibit the sale of tobacco from vending machines, a significant source of tobacco for young people, subject to Parliamentary consideration of regulations.	R	L	2
4. Take action to ensure that the advertising of tobacco accessories is not being used to encourage the use of tobacco products of any type.	V	L	2
Encourage research to further our understanding of the possible links between tobacco packaging and smoking behaviours.			
5. Restrict tobacco availability to children by reviewing the current restrictions on the retail of tobacco and enforcement of tobacco retail regulations. Alongside this, we will launch a review within the first three months of this year into the purchase for and supply of tobacco to young people. This review will assess what more can be done to limit these sources, including examining the current legislation around the confiscation of tobacco that children are found to have in their possession.	R,C	L,R	2

6. Continue to engage with young people to raise awareness about the dangers of smoking and develop skills that will encourage them to play a role in building our smokefree future.	E,T	C,S	1.5
**Motivating and helping smokers to stop**			
7. Continue to deliver marketing campaigns to encourage more quit attempts, particularly targeted at smokers from more disadvantaged backgrounds.	P	C	1.5
8. Support primary care trusts (PCTs) to increase the percentage of smokers successfully supported by NHS Stop Smoking Services to quit.	N	S	2
Introduce a radical approach to quitting smoking, producing more routes to quitting that we believe will help thousands more smokers quit successfully, particularly among disadvantaged communities where evidence suggests that smokers are more addicted. Although always encouraging smokers to break their nicotine dependence entirely, the new routes will support those smokers who are unable to quit abruptly to: cut down their levels of smoking as a precursor to completely quitting; manage their nicotine addiction, using a safer alternative product, when they are unable to smoke (e.g. at work); and dramatically reduce the damage to their health, and the harms to those around them, by using a safer alternative to smoking.	n/a	n/a	
9. Deliver this new approach with the Medicines and Healthcare products Regulatory Agency (MHRA) encouraging the development, marketing and wide availability of nicotine delivery medicines. To create a level playing field the MHRA will consult on regulating all nicotine-containing products (with the exception of tobacco products, which are governed through specific legislation), and a consultation on how this level playing field should be created is being launched alongside this strategy.	N	R	1.5
10. Support the creation of new routes to quitting by working with PCTs to develop a model for a new type of NHS smoking-cessation service that will support smokers who use the new routes to quitting.	N	S	1
**Protecting non-smokers**			
11. Promote ‘smokefree communities’ through awareness campaigns and projects focused on the most disadvantaged communities that highlight the benefits of smokefree homes and cars. We will do this working in alliance with public sector and voluntary organisations, private businesses, civil society and local government to protect children from secondhand smoke.	E,P	C	1.5
12. Consider as part of a review of the Health Act 2006 what further action is needed to protect people, including support for the extension of smokefree around doorways and protection for children from secondhand smoke exposure.	R	L	2
Encourage and celebrate a tobacco-free London 2012 Olympics without the use or sale of tobacco at Olympic venues.			
Support PCTs to work through local partnerships to develop public health interventions that consider lifestyle factors in the whole and consider the social, economic and cultural factors that influence smoking rates.			
Develop more effective methods of identifying and			

preventing smoking in pregnancy.			
Total agreement/total possible			21/24

The NICE Obesity Guidelines (2006)

Context: This is the first national guidance on the prevention, identification, assessment and management of overweight and obesity in adults and children in England and Wales. The guidance aims to:

- stem the rising prevalence of obesity and diseases associated with it
- increase the effectiveness of interventions to prevent overweight and obesity
- improve the care provided to adults and children with obesity, particularly in primary care.

Coding Key:
Interventions
Education E, Persuasion P, Incentivisation I, Coercion C, Training T, Restriction R, Environmental restructuring V, Modelling M, Enablement/resources N, Unclassifiable U
Policies
Fiscal F, Communication/marketing C, Service provision S, Legislation L, Regulation R, Guidelines G, Environmental/social planning E, Unclassifiable U

This Table: shows the agreed intervention function/s and policy category/ies along with the number for which there was agreement and/or disagreement.

**NICE Obesity Guidance, (2006): Key priorities for implementation**	**Intervention function**	**Policy category**	**Agreement (n)**	**Disagree (n)**
**Public health**				
NHS				
• Managers and health professionals in all primary care settings should ensure that preventing and managing obesity is a priority, at both strategic and delivery levels. Dedicated resources should be allocated for action.	V	U	0	2
Local authorities and partners				
• Local authorities should work with local partners, such as industry and voluntary organisations, to create and manage more safe spaces for incidental and planned physical activity, addressing as a priority any concerns about safety, crime and inclusion, by:	V	E	2	0
– providing facilities and schemes such as cycling and walking routes, cycle parking, area maps and safe play areas	V	E	2	0
– making streets cleaner and safer, through measures such as traffic calming, congestion charging, pedestrian crossings, cycle routes, lighting and walking schemes	V	E	2	0
– ensuring buildings and spaces are designed to encourage people to be more physically active (for example, through positioning and signing of stairs, entrances and walkways)	V	E	2	0
– considering in particular people who require tailored information and support, especially inactive,	E, N	U	2	1

vulnerable groups.				
Early years settings				
• Nurseries and other childcare facilities should:				
– minimise sedentary activities during play time, and provide regular opportunities for enjoyable active play and structured physical activity sessions	N	U	2	0
– implement Department for Education and Skills, Food Standards Agency and Caroline Walker Trust[1] guidance on food procurement and healthy catering.	U	G	2	0
Schools				
• Head teachers and chairs of governors, in collaboration with parents and pupils, should assess the whole school environment and ensure that the ethos of all school policies helps children and young people to maintain a healthy weight, eat a healthy diet and be physically active, in line with existing standards and guidance. This includes policies relating to building layout and recreational spaces, catering (including vending machines) and the food and drink children bring into school, the taught curriculum (including PE), school travel plans and provision for cycling, and policies relating to the National Healthy Schools Programme and extended schools.	V,E,N, R	G	7	2
Workplaces				
• Workplaces should provide opportunities for staff to eat a healthy diet and be physically active, through:				
– active and continuous promotion of healthy choices in restaurants, hospitality, vending machines and shops for staff and clients, in line with existing Food Standards Agency guidance	V,N,P	V,G,C	3	3
– working practices and policies, such as active travel policies for staff and visitors	U	G	2	0
– a supportive physical environment, such as improvements to stairwells and providing showers and secure cycle parking	V	E	2	0
– recreational opportunities, such as supporting out-of-hours social activities, lunchtime walks and use of	N	G	2	0

[1]see www.cwt.org.uk

local leisure facilities.				
Self-help, commercial and community settings				
• Primary care organisations and local authorities should recommend to patients, or consider endorsing, self-help, commercial and community weight management programmes only if they follow best practice (see recommendation 1.1.7.1 for details of best practice standards).	P	G	2	0
**Clinical care**				
Children and adults				
• Multicomponent interventions are the treatment of choice. Weight management programmes should include behaviour change strategies to increase people's physical activity levels or decrease inactivity, improve eating behaviour and the quality of the person's diet and reduce energy intake.	N	S	0	2
Children				
• Interventions for childhood overweight and obesity should address lifestyle within the family and in social settings.	U	U	2	0
• Body mass index (BMI) (adjusted for age and gender) is recommended as a practical estimate of overweight in children and young people, but needs to be interpreted with caution because it is not a direct measure of adiposity.	U	U	2	0
• Referral to an appropriate specialist should be considered for children who are overweight or obese and have significant comorbidity or complex needs (for example, learning or educational difficulties).	N	S	2	0
Adults				
• The decision to start drug treatment, and the choice of drug, should be made after discussing with the patient the potential benefits and limitations, including the mode of action, adverse effects and monitoring requirements and their potential impact on the patient's motivation. When drug treatment is prescribed, arrangements should be made for appropriate health professionals to offer information, support and counselling on additional diet, physical activity and behavioural strategies. Information about patient support programmes should also	E,N	S	2	2

be provided.				
• Bariatric surgery is recommended as a treatment option for adults with obesity if all of the following criteria are fulfilled:	N	S	2	0
• Bariatric surgery is also recommended as a first-line option (instead of lifestyle interventions or drug treatment) for adults with a BMI of more than 50 kg/m^2 in whom surgical intervention is considered appropriate.	N	S	2	0
Totals			44	12
